# Site-Specific Antioxidative Therapy for Prevention of Atherosclerosis and Cardiovascular Disease

**DOI:** 10.1155/2013/796891

**Published:** 2013-04-30

**Authors:** Hajime Otani

**Affiliations:** Second Department of Internal Medicine, Kansai Medical University, Moriguchi City 570-8507, Japan

## Abstract

Oxidative stress has been implicated in pathophysiology of aging and age-associated disease. Antioxidative medicine has become a practice for prevention of atherosclerosis. However, limited success in preventing cardiovascular disease (CVD) in individuals with atherosclerosis using general antioxidants has prompted us to develop a novel antioxidative strategy to prevent atherosclerosis. Reducing visceral adipose tissue by calorie restriction (CR) and regular endurance exercise represents a causative therapy for ameliorating oxidative stress. Some of the recently emerging drugs used for the treatment of CVD may be assigned as site-specific antioxidants. CR and exercise mimetic agents are the choice for individuals who are difficult to continue CR and exercise. Better understanding of molecular and cellular biology of redox signaling will pave the way for more effective antioxidative medicine for prevention of CVD and prolongation of healthy life span.

## 1. Introduction

 Oxidative stress has been implicated in pathophysiology of aging and age-associated disease. Reactive oxygen species (ROS) have been considered as harmful molecules for organisms by destroying DNA and cell structures, thereby deteriorating multiple organ function, leading to aging. However, recently emerging paradigm reinforces a novel role of ROS as a messenger of redox signaling that modulates aging processes [1]. Perturbation of the redox signaling due to oxidative stress deteriorates endothelial function and promotes atherosclerosis. Thus, elucidation of the origin of ROS and the mechanism of ROS generation in endothelial cells are pivotal to develop effective strategies for prevention of atherosclerosis, aging, and cardiovascular disease (CVD).

The major origins of ROS in endothelial cells are mitochondrial electron transfer chain (ETC), NADPH oxidase (Nox), endothelial nitric oxide synthase (eNOS), and xanthine oxidase (XO). Mitochondrial ETC inevitably generates ROS associated with oxidative phosphorylation and energy production. The amount of ROS production by mitochondria increases with age and under certain pathophysiological conditions such as excessive food intake and sedative lifestyle [2, 3]. Besides this intrinsic mechanism of oxidative stress, there are extrinsic mechanisms of oxidative stress that enhances ROS generation by stimulating ROS generating machinery within endothelial cells. Endothelial Nox activity is known to be increased by proinflammatory cytokines [4]. Visceral adipose tissue is a main source of proinflammatory cytokines such as tumor necrosis factor-*α* and interleukin-6 in individuals with abdominal obesity [5, 6]. Proinflammatory adipocytokines contribute to ROS generation and endothelial dysfunction through upregulation of Nox, leading to insulin resistance or type 2 diabetes (DM), hypertension, and a variety of CVDs. Local activation of renin-angiotensin system (RAS) also contributes to the enhanced expression and activation of Nox [7, 8]. Nox-derived ROS then promotes uncoupling of eNOS and exaggerates oxidative stress and endothelial dysfunction [9–11]. Endothelial dysfunction by any causes including cigarette smoking, stimulation with angiotensin II, or inflammatory cytokines results in activation of xanthine oxidase and further production of ROS [12, 13]. Thus, once ROS generation overwhelms the antioxidative capacity, oxidative stress propagates by creating a self-perpetuating cycle and accelerates endothelial dysfunction and atherosclerosis.

Extensive efforts have been exerted to ameliorate oxidative stress in the cardiovascular system especially to endothelial cells by general antioxidants. However, these antioxidants have conferred only limited success to prevent CVD. On the other hand, a growing body of evidence suggests that the site-specific blockade of ROS production might represent an alternative strategy to prevent atherosclerosis and CVD. The present review will discuss the issue as to why general antioxidants have failed to provide appreciable antiatherosclerotic effects, and how the site-specific antioxidative therapy exerts beneficial effects on the cardiovascular system. 

## 2. Effects of General Antioxidants on Cardiovascular Disease

The use of general antioxidants has become a common practice for prevention of CVD and age-associated disease. However, there are as yet no clinical indications for the routine use of antioxidants for treatment of these diseases. This is because no appreciable benefits have been demonstrated in multiple clinical trials that employed general antioxidants. For example, a large trial of vitamin E and *β*-carotene failed to show any protective cardiovascular effects when smokers with acute myocardial infarction (MI) were treated for a long term with these agents [14]. Conversely, this clinical study revealed that the risk of fatal CVD increased in the groups that received either *β*-carotene or the combination of vitamin E and *β*-carotene. Some explanations have been offered to give an insight into the failure of clinical studies using general antioxidant therapies. It may be argued that more than one antioxidant is required for clinical effectiveness. This idea is based on the assumption that antioxidants exist as a “network,” wherein both lipid soluble (like vitamin E) and water soluble (ascorbate, glutathione, and dihydrolipoic acid) molecules work in a network for the removal of oxidative stress in conjunction with the regeneration of oxidant defenses. To date clinical trials have generally not used synergistic combinations of lipid soluble and water soluble antioxidants despite the theoretical advantages and basic science demonstrations of effectiveness of “antioxidant cocktail.” Another explanation for the failure of clinical antioxidant studies is that antioxidants can act as prooxidants by retaining high energy electrons when scavenging ROS or reducing iron. It is also possible that antioxidants or by-products may impair mitochondrial oxidative phosphorylation and ATP production. For example, the *β*-carotene cleavage products have been shown to strongly inhibit state-3 respiration in isolated liver mitochondria [15]. Therefore, it is desirable for antioxidants to scavenge ROS without unfavorable side effects to effectively eliminate oxidative stress.

Astaxanthin, a xanthophyll carotenoid, which is contained in a variety of seafoods such as crabs, crayfish, krill, lobsters, salmon, shrimp, and trout, may represent a novel class of general antioxidant. Unlike other general antioxidants, astaxanthin is not converted into a prooxidant when scavenging ROS. Astaxanthin neutralizes ROS or other oxidants by either accepting or donating electrons, and without being destroyed or becoming a prooxidant in the process, because its linear, polar-nonpolar-polar molecular layout equips it to precisely insert into the membrane and span its entire width. In its position spanning the membrane, astaxanthin can intercept reactive molecular species within the membrane's hydrophobic interior and along its hydrophilic boundaries, thereby providing versatile antioxidant actions. 

Diverse benefits of astaxanthin in the clinical arena, with excellent safety and tolerability, have been extensively reviewed [16]. It appears that astaxanthin clinical success extends beyond protection against oxidative stress and inflammation to promise for slowing age-related functional decline. Nevertheless, larger sized randomized controlled trials for subjects with lifestyle-related diseases are required to address a question whether astaxanthin prevents CVD and the related mortality, which are the most important clinical end point. 

## 3. Effects of General Antioxidants on Ischemic Tolerance

The use of general antioxidants may not be an ideal choice for treatment of CVD especially in subjects at high risk of MI or cerebral infarction. Indiscriminate removal of ROS may reduce the tolerance to hypoxic or ischemic stress, which may worsen the prognosis of heart attack. It is known that ROS is necessary for acquisition of tolerance to lethal ischemic insult. The most representative example of this phenomenon is ischemic preconditioning (IPC). IPC was first discovered by Murry and associates [17] who demonstrated that a brief period of repetitive cardiac ischemia/reperfusion exerts a protective effect against subsequent lethal periods of ischemia. It is now evident that IPC has two distinct phases: an early phase, which lasts from a few minutes to 2-3 hours and a late phase, termed late IPC, which develops after 12 hours, peaks between 24 and 48 hours, and lasts for 72–96 hours [18]. Recent advances in preconditioning research at molecular and cellular levels suggest that cardioprotective signal transduction in early IPC proceeds through a self-perpetuating cycle of redox signaling including activation of protein kinase C-*ε* and phosphatidylinositol-3 kinase which culminates in protection of mitochondria against ROS- and Ca^2+^-induced opening of mitochondrial permeability transition pore through activation of mitochondrial K_ATP_ channels and inhibition of glycogen synthase kinase-3*β* [19]. Positive feedback and feedforward amplification of redox signaling induced by activation of mitochondrial K_ATP_ channels plays a crucial role in developing the memory of cardioprotection that converges on mitochondria. 

ROS generated during brief ischemia and reperfusion cycles have been consistently implicated in the trigger of IPC [20–22]. In contrast to detrimental effects by massive generation of ROS, sublethal amounts of ROS could serve as a trigger of IPC. Because IPC is implemented by pretreatment with single or multiple brief periods (<10 min) of ischemia and reperfusion prior to more prolonged and potentially lethal period of ischemia, it is conceivable that IPC procedures generate relatively small amounts of ROS compared with a lethal period of ischemia and reperfusion. Such ROS production could function as a messenger of signaling cascades to protect against lethal oxidative stress induced by a subsequent prolonged period of ischemia and reperfusion by inhibiting robust increase in ROS generation in mitochondria [23]. In addition, redox signaling may be a universal feature of cardiomyocyte response to all forms of stress such as hyperthermia and a mechanism for acquisition of ischemic tolerance [24]. Therefore, antioxidant medicine must be more site specific, being targeted to a specific ROS or cellular compartment, without a deleterious effect on favorable redox-sensitive signaling pathways. 

## 4. Site-Specific Inhibition of NADPH Oxidase

### 4.1. Blockers of the Renin-Angiotensin System

Nox is a major ROS generating enzyme in the cardiovascular system. It has been demonstrated that Nox-derived ROS plays a physiological role in the regulation of endothelial function and vascular tone and a pathophysiological role in atherosclerosis and angiogenesis. There are seven homologs of Nox (Nox1, Nox2, Nox3, Nox4, Nox5, Duox1, and Duox2) [25]. Nox2 also known as gp91^phox^ was originally found in phagocytes such as neutrophils and macrophages. Nox2 plays a crucial role in host defense against microorganisms by releasing ROS upon the respiratory burst. The absence of Nox2 gene causes repetitive infections and premature death known as chronic granulomatous disease at an early stage of life [26]. Thus, indiscriminate inhibition of Nox2 abrogates bactericidal capacity of the phagocytes. Nox2 is inducible in cardiovascular systems such as in endothelial cells, vascular smooth muscle cells (SMCs), and cardiomyocytes by stimulation with inflammatory cytokines and angiotensin II [27, 28]. Nox1, which is the first homolog of Nox2, is also activated in endothelial cells under the pathological conditions that lead to atherosclerosis [29]. Conversely, Ray et al. [30] demonstrated that, unlike Nox1 or Nox2, Nox4 is constitutively activated in endothelial cells without stimulation with inflammatory cytokines or angiotensin II and acts as a vasodilator through H_2_O_2_-induced hyperpolarization of endothelial cells. These findings indicate that the use of nonselective Nox inhibitors is not suitable for ameliorating oxidative stress in the cardiovascular system and provides a basis for employing specific inhibitors that block only inducible Nox.

Some of the recently emerging drugs for CVD possess a property to inhibit inducible Nox as a preferable side effect. Angiotensin converting enzyme inhibitors (ACEs) and angiotensin II type 1 (AT1) receptor blockers (ARBs) are such a class of antihypertensive drugs. A growing body of evidence suggests that enhanced activation of the RAS is a key to promote endothelial dysfunction and hypertension [31, 32]. 

Nox plays a crucial role in the RAS-mediated development of atherosclerosis. Nox-derived ROS oxidizes LDL that is incorporated into the intima of the blood vessel. Oxidized LDL is then taken up by macrophages via their scavenger receptors CD36 to form foam cells. Macrophage recruitment to the intima is likely to be regulated not only by a multiplicity of adhesion molecules, integrins, and selectins, but also by chemokines such as macrophage chemoattractant protein-1, which is constitutively synthesized and secreted by endothelial cells and SMCs migrated from the media [33, 34]. Transcriptional upregulation of these molecules is enhanced by ROS, which are derived at least in part from Nox in endothelial cells and SMCs, creating a self-amplifying loop in foam cell accumulation and atherosclerotic plaque formation. 

Local activation of the RAS in the myocardium induced by hypertension and after myocardial infarction plays a crucial role in pathological remodeling of the heart. The RAS is activated in response to mechanical or hypoxic stress to cardiomyocytes under these conditions. AT1 receptor stimulation induces oxidative stress on cardiomyocytes by activating Nox, which triggers cardiomyocyte hypertrophy by stimulating hypertrophy signaling such as protein kinase C and ERK [35, 36]. AT1 receptors are also known to activate transforming growth factor-*β* signaling and promote interstitial fibrosis [37]. Cardiomyocyte hypertrophy and interstitial fibrosis are the two important events for transition to heart failure from physiological adaptation against pressure overload to the heart. 

There is another reason for the preferential use of ACE inhibitors and ARBs for hypertensive subjects with visceral obesity. Adipocytes in the visceral adipose tissue are a suggested source of components of the RAS, with regulation of their production related to obesity-induced hypertension. Angiotensin II has been demonstrated to promote oxidative stress via overexpression of Nox in adipocytes. It has been demonstrated that blockade of AT1 receptors reduces oxidative stress in adipose tissue and ameliorates production of proinflammatory cytokines responsible for systemic inflammation and oxidative stress [38]. Thus, ACEs and ARBs are capable of inhibiting Nox which is upregulated in the cardiovascular system and the visceral adipose tissue by local activation of the RAS. Importantly, ACEs and ARBs do not affect Nox activity in cells without activation of the RAS.

### 4.2. Inhibitors of HMG-CoA Reductase

Because atherosclerosis is facilitated by dyslipidemia in conjunction with oxidative stress, numerous studies have investigated relative contribution of dyslipidemia and oxidative stress to atherogenesis. It has been demonstrated that a 3-hydroxy-3-methylglutaryl CoA reductase inhibitor lovastatin significantly reduced total cholesterol, selectively decreased non-HDL-cholesterol, and significantly reduced fatty streak lesion formation in the aortic arch of the diabetic hamsters fed the atherogenic diet [39]. In this study, while vitamin E and probucol were effective in reducing several indices of oxidative stress including plasma lipid peroxides, cholesterol oxidation products, and in vitro LDL oxidation, they had no effect on fatty streak lesion formation. It appears that in this combined model of type 2 DM and hypercholesterolemia, lovastatin prevented progression of fatty streak lesion formation by reducing total cholesterol and non-HDL-cholesterol and inhibiting oxidative stress.

The pleiotropic effects of statins that prevent oxidative stress and atherosclerosis have been extensively investigated. Emerging evidence suggests that these cholesterol-independent effects are predominantly due to their ability to inhibit isoprenoid synthesis, particularly geranylgeranylpyrophosphate and farnesylpyrophosphate, which are important posttranslational lipid attachments of the Rho GTPases and activation of its downstream target, Rho-kinase (ROCK) [40]. Angiotensin II-induced activation of Nox requires Rho GTPase association with Nox [41, 42]. Inhibition of ROCK by statins may also be associated with inhibition of oxidative stress mediated by activation of Nox. It has been shown that rosuvastatin attenuated the angiotensin II-mediated upregulation of Nox subunits associated with downregulation of AT1 receptors and the lectin-like oxidized LDL receptor LOX-1, leading to the reduction of oxidized LDL [43]. In this regard, improvement of endothelial dysfunction, cerebral ischemia and coronary vasospasms by statin inhibition of ROCK is also attributed to upregulation of eNOS, which decreases vascular inflammation and reduces atherosclerotic plaque formation [44]. 

### 4.3. Exercise

A number of studies show that increased physical activity reduces oxidative stress. The mechanism underlying reduced oxidative stress by exercise may be related to the decreased expression of Nox in the visceral adipose tissue associated with the prevention of dysregulated production of inflammation-related adipocytokines, suggesting that exercise is a fundamental approach to protect against cardiovascular risk. Szostak and Laurant [45] have proposed that exercise promotes atheroprotection possibly by reducing or preventing oxidative stress and inflammation through at least two distinct mechanisms. Exercise, through laminar shear stress activation, downregulates endothelial AT1 receptor expression, leading to decreases in Nox activity and ROS generation, and preserves endothelial NO bioavailability and its protective antiatherogenic effects. These observations suggest that lifestyle modification can prevent Nox activation and cardiovascular oxidative stress without relying on ARBs, ACEs, or statins.

## 5. Site-Specific Inhibition of Xanthine Oxidase

 A substantial body of experimental and epidemiological evidence suggests that serum uric acid is an important and independent risk factor for cardiovascular and renal disease, especially in patients with heart failure, DM, and hypertension [46]. The level of uric acid in the blood is increased with age, body mass index, and hypertension [47]. Hyperuricemia is caused by excessive intake of dietary purine or ethanol, activation of xanthine oxidase (XO), and decreased renal excretion of uric acid. Of these, XO, which metabolizes xanthine as a substrate, is intimately related to endothelial dysfunction. XO is converted from xanthine dehydrogenase in endothelial cells by oxidative stress or stimulation with inflammatory cytokines or angiotensin II [48]. 

A large body of evidence supports an important role for XO-mediated ROS generation in atherosclerosis [49–51]. Landmesser et al. [52] demonstrated that angiotensin II substantially increased endothelial XO protein levels and XO-dependent superoxide production in cultured endothelial cells, which was prevented by Nox inhibition. Atherosclerosis is prone to be produced in the bifurcation of blood vessels associated with oscillatory shear stress. It has been demonstrated that conversion of XO from xanthine dehydrogenase and resultant ROS production are increased by oscillatory shear stress in an Nox-dependent manner in endothelial cells [53]. These observations suggest that conversion of XO from xanthine dehydrogenase is dependent on angiotensin II-mediated activation of Nox in endothelial cells, and XO plays a crucial role in angiotensin II-induced development of atherosclerosis. XO-derived ROS is also involved in SMC proliferation and death, which are the important steps for atherosclerosis. Interestingly, a single exposure of cultured rat vascular SMCs to XO/xanthine predominantly resulted in cell proliferation, whereas frequent exposures to high levels of XO/xanthine predominantly resulted in cell death by apoptosis [54]. Thus, inhibition of XO is a promising approach to prevent atherosclerosis.

XO mediates tissue injury during reperfusion typically encountered in the heart after percutaneous coronary interventions for acute MI or open-heart surgery. It has been demonstrated that XO in the heart is activated by tumor necrosis factor (TNF)-*α*, which is upregulated after ischemia/reperfusion, and administration of anti-TNF-*α* antibodies at the onset of reperfusion partially restores nitric oxide- (NO-) mediated coronary arteriolar dilation and reduces superoxide production [55, 56]. These studies suggest that anticytokine therapy after reperfusion may be effective in ameliorating reperfusion injury by preventing activation of XO. However, the effect of anti-TNF-*α* antibodies against ischemia/reperfusion injury remains controversial, presumably by the fact that TNF-*α* also mediates cardioprotective signal transduction as observed in IPC [57].

Allopurinol and its active metabolite oxypurinol are the representative inhibitors of XO. Inhibition of XO by allopurinol is known to prevent atherosclerosis. It has been demonstrated that allopurinol reduces neointimal hyperplasia in the carotid artery ligation model in spontaneously hypertensive rats [58]. In addition, oral administration of allopurinol to ApoE knockout mice markedly ameliorated oxidized LDL accumulation and calcification in the aorta and aortic root [59]. The antiatherosclerotic effect of allopurinol has also been reported in clinical studies for patients with a variety of cardiovascular risk factors. Long-term and high-dose allopurinol therapy significantly improved endothelial function in diabetic normotensive patients [60]. Allopurinol reversed endothelial dysfunction in smokers [13]. Furthermore, there is evidence that allopurinol improves endothelium-dependent vascular function in patients with chronic heart failure [61].

It has been established that allopurinol protects reperfusion injury of the heart. Manning et al. [62] for the first time demonstrated that reperfusion-induced arrhythmias and infarct size are reduced by allopurinol in rats. Similar findings were reported by a number of other investigators using allopurinol or oxypurinol in diverse animal models such as rat, rabbit, dog, and pig [46]. The mechanism of allopurinol prevention of myocardial reperfusion injury seems to be provoked by protection of endothelial cells from oxidative stress. Gao et al. [56] demonstrated that administration of allopurinol at the time of reperfusion reduced superoxide production and ameliorated coronary endothelial dysfunction. Taken together, these findings suggest that specific inhibition of XO confers protection against ischemia/reperfusion injury.

## 6. Site-Specific Inhibition of Nitric Oxide Synthase Uncoupling

NO plays a central role in the regulation of cardiovascular function. NO is one of gaseous signaling molecules, which were previously considered to be toxic. However, the identification of NO as the endothelium-derived relaxing factor combined with the discovery of NO generation by NOS primed an explosion of research in this area in the 1990s [63–65]. It is now evident that NO and cognate reactive nitrogen intermediates are involved in a wide variety of pathophysiological processes in the cardiovascular system where it orchestrates a plethora of cellular activities in cardiomyocytes, endothelial cells, vascular SMCs, and circulating inflammatory cells.

Biosynthesis of NO is dependent on enzymatic activity of NOS. NOS is a homodimeric oxidoreductase containing iron protoporphyrin IX (heme), flavin adenine dinucleotide, flavin mononucleotide, and tetrahydrobiopterin (BH_4_) which is a cofactor essential for the catalytic activity of all three NOS isoforms [66, 67]. The flavin-containing reductase domain and a heme-containing oxygenase domain are connected by a regulatory calmodulin-binding domain. In the case of constitutive NOS, that is, nNOS and eNOS, binding of Ca^2+^/calmodulin orients the other domains to allow NADPH-derived electrons generated in the reductase domain to flow to the oxygenase domain [68], ultimately resulting in the conversion of L-arginine to NO and L-citrulline. This occurs if BH_4_ is bound in the dimer interface, where it interacts with amino acid residues from both monomers to stabilize NOS dimerization and participate in arginine oxidation through the N-hydroxyl-L-arginine intermediate and the subsequent generation of NO. BH_4_ depletion because of its oxidation and/or reduced synthesis can result in functional uncoupling of NOS. Uncoupled NOS generates more ROS and less NO, shifting the nitroso-redox balance and having adverse consequences on the cardiovascular system [10]. Thus, modulation of the arginine-NO pathway by BH_4_ may be beneficial for prevention of CVD.

 BH_4_ is highly sensitive to oxidation by ROS and peroxynitrite and is converted to dihydrobiopterin (BH_2_). Oxidative stress imposed on endothelial cells causes depletion of BH_4_ and eNOS uncoupling. The effect of eNOS uncoupling has been investigated in a wide variety of in vitro models, animal models of CVD, and human subjects with cardiovascular risk factors [10, 69, 70]. Inhibition of oxidative stress to endothelial cells and subsequent occurrence of NOS uncoupling provide an ameliorative effect on endothelial function. Therefore, any interventions against oxidative stress to endothelial cells such as lifestyle modification, especially calorie restriction and endurance exercise, or any pharmacological tools such as the use of ARBs, statins, and xanthine oxidase inhibitors, may represent a promising approach to the prevention of NOS uncoupling.

In many cases, supplementation with BH_4_ under pathological conditions with oxidative stress has been shown to reverse eNOS dysfunction. However, true mechanistic relationship between endothelial BH_4_ levels and eNOS regulation in vivo by administration of BH_4_ remains controversial. High extracellular BH_4_ concentrations may result in nonspecific antioxidant effects that indirectly increase NO bioactivity by scavenging ROS rather than by modulating eNOS activity. Furthermore, the effects of supplementation with BH_4_ or biopterin analogues on NO bioactivity are unpredictable in vascular disease states in which oxidative stress is increased [71, 72]. Indeed, it remains unclear whether adequate eNOS cofactor function in vivo is related to absolute BH_4_ levels in the endothelial cell, or whether the relative balance between reduced BH4 and oxidized BH_2_ may be more important [73]. 

Intracellular BH_4_ levels are regulated by the activity of the de novo biosynthetic pathway and the salvage pathway. In the de novo biosynthetic pathway guanosine triphosphate cyclohydrolase (GTPCH)-1 catalyzes GTP to dihydroneopterin triphosphate. BH_4_ is generated by further steps catalyzed by 6-pyruvoyltetrahydropterin synthase and sepiapterin reductase. GTPCH-1 appears to be the rate-limiting enzyme in BH_4_ biosynthesis; transgenic overexpression of GTPCH-1 is sufficient to augment BH_4_ levels in endothelial cells and preserve NO-mediated endothelial function in diabetic mice. In the salvage pathway, BH_4_ is synthesized from BH_2_ by sepiapterin reductase and dihydrofolate reductase [74]. Exogenous BH_4_ is labile in physiological solution. It has been reported that in vivo half-life of BH_4_ is 3.3–5.1 hour in the plasma of healthy adult humans [75]. Because not all oxidized BH_4_ is converted to BH_2_, which is further degraded to dihydroxanthopterin and excreted to urine [76], BH_2_ availability for the salvage pathway may be limited under oxidative stress even with BH_4_ supplementation. Thus, sepiapterin may serve as an effective substrate for BH_4_ via the salvage pathway. Folic acid and vitamin C are also able to restore eNOS functionality, most probably by enhancing BH_4_ levels through mechanisms yet to be clarified [76]. 

The effect of BH_4_ on CVD has been investigated in various animal models and in human subjects. BH_4_ ameliorated endothelial dysfunction and reversed hypoadiponectinemia as a result of oxidative stress in rats [77]. In addition, intramuscular GTPCH-1 gene transfer using atelocollagen was found to serve as a useful method of long-term systemic delivery of BH_4_ and the treatment of endothelial dysfunction in insulin-resistant rats [76, 78]. The therapeutic efficacy of BH_4_ has been examined in patients with hypertension, peripheral arterial disease, and coronary artery disease, and these studies consistently demonstrated the beneficial effect of BH_4_ on endothelial dysfunction [79]. BH_4_ also improved endothelium-dependent vasodilation in chronic smokers [80]. However, a phase 2 clinical trial sponsored by the US pharmaceutical company BioMarin failed to observe an ameliorative effect of oral administration of BH_4_ in patients with poorly controlled hypertension. This finding is not surprising, because chronic exposure to NO causes nitrate tolerance through *S*-nitrosylation and desensitization of soluble guanylyl cyclase, an enzyme that generates cGMP which is largely attributed to the vasodilating effect of NO [81]. Although long-lasting treatment with BH_4_ may not be effective in ameliorating hypertension through an NO/cGMP-dependent mechanism, recent studies suggest that an NO-dependent *S*-nitrosylation plays a key role in posttranscriptional modification of the variety of key proteins involved in cardiac contractile function and antiarrhythmias, angiogenesis, and protection against ischemia/reperfusion injury [82–86]. These reports lend support to the hypothesis that supplementation with BH_4_ may represent a promising therapeutic strategy for heart failure and ischemic heart disease. Further studies are warranted to address whether BH_4_ or its precursors truly exert salutary effects on endothelial dysfunction in a variety of CVD.

## 7. Strategies to Prevent Mitochondrial Generation of ROS

### 7.1. Stimulation of Mitochondrial Biogenesis

 Mitochondria are the critical component in control of aging. Mitochondrial dysfunction and increased generation of ROS are a prominent feature of aging and various age-related neurodegenerative diseases such as Parkinson's disease and Alzheimer disease [87]. Mitochondrial dysfunction has also been implicated in CVD [88]. The heart has the highest oxygen uptake in the human body, and accordingly it has a large number of mitochondria. This high dependence on mitochondrial metabolism has its costs: when oxygen supply is threatened, high leak of electrons from the ETC leads to oxidative stress and mitochondrial failure. Indeed, it is estimated that, of the oxygen consumed by mammalian cells, 0.2–2% of it is converted to ROS, and most of the ROS have mitochondrial origins [89, 90]. 

Mitochondrial generation of ROS is increased with age due to a decrease in electron transport function especially at the site of complex I in the ETC [91]. This leads to stagnation of electrons in the ETC and increases leakage of electrons that react with oxygen to generate superoxide. Stagnation of electrons is augmented by increased influx of electrons to the ETC due to excessive food intake or reduced physical activity [92, 93]. The elevation of ATP/ADP ratio by excessive food intake or reduced physical activity also enhances stagnation of electrons and the generation of ROS in the ETC by decreasing the passage of protons through ATP synthase [94].

Improvement of ETC function may be the most effective approach to reduce the production of ROS in mitochondria. An increase in mitochondrial biogenesis has been shown to improve ETC function and reduce ROS production. A growing body of evidence suggests that AMP-activated protein kinase (AMPK) plays a pivotal role in mitochondrial biogenesis [95]. AMPK acts as a cellular energy sensor that is activated by a decrease in ATP/AMP ratio [96]. The decrease in ATP/AMP ratio is most commonly induced by calorie restriction (CR) and endurance exercise. CR and endurance exercise have advantageous effects on blood pressure level in humans and also beneficially influence blood lipid profile and glucose homeostasis in individuals displaying features of metabolic syndrome [97]. Thus, CR and endurance exercise may enhance mitochondrial biogenesis through common signaling pathways. The AMPK-mediated increase in mitochondrial biogenesis occurs through the activation of SIRT1, which can mimic several metabolic aspects of CR by targeting selective nutrient utilization and mitochondrial oxidative function to regulate energy balance [98]. SIRT1 then activates peroxisome proliferator-activated receptor-*γ* coactivator (PGC)-1*α* through deacetylation reaction. PGC-1*α* is an end target of events by which AMPK promotes mitochondrial biogenesis. Valle et al. [99] have demonstrated that endothelial cells that overexpress PGC-1*α* show reduced accumulation of ROS, increased mitochondrial membrane potential, and reduced apoptotic cell death both in basal and oxidative stress conditions. 

The mechanism by which AMPK upregulates PGC-1*α* appears to be triggered by mitochondrial generation of ROS. Irrcher et al. [100] demonstrated that AMPK activation in the presence of H_2_O_2_ increased PGC-1*α* promoter activity with concomitant increases in mRNA expression. This observation suggests that mitochondria-derived ROS is necessary for AMPK and SIRT1 to upregulate PGC-1*α* that feedbacks to inhibit mitochondrial generation of ROS by increasing mitochondrial biogenesis. Thus, AMPK upregulation of PGC-1*α* is another example for redox signaling-dependent protection of mitochondria.

eNOS-derived NO and AMPK synergistically increase the activity of PGC-1*α*. NO induces mitochondrial biogenesis in skeletal muscle cells via upregulation of PGC-1*α* [101]. Similar effects of NO on PGC-1*α* and mitochondrial biogenesis were also observed in fibroblasts and adipocytes [102]. Thus, NO-induced maintenance of metabolic function and prevention of oxidative stress is at least in part mediated by AMPK and PGC-1*α*.

Nuclear respiratory factor (NRF) is another redox-sensitive transcriptional factor that is activated in the presence of ROS derived from mitochondria [103, 104]. Mitochondrial biogenesis depends on the interplay between NRF and PGC-1*α* [105]. NRF is, therefore, an emerging target to counteract mitochondrial dysfunction and ROS generation. NRF mediates the biogenomic coordination between nuclear and mitochondrial genomes by directly regulating the expression of several nuclear-encoded ETC proteins through nuclear mitochondrial interactions with PGC-1*α* [106]. Taken together, these findings suggest complex interplay between AMPK, SIRT1, PGC-1*α*, and NRF under the redox regulation upon CR and endurance exercise.

 Because the long-term adoption of a CR lifestyle is not realistic in a significant proportion of the human population, the search for substances that can reproduce the beneficial physiologic responses of CR without a requisite calorie intake reduction, termed CR mimetic agents, have gained momentum. Of these, CR mimetics that activate NRF and PGC-1*α*, thereby promoting mitochondrial biogenesis and ameliorating oxidative stress, may represent a promising pharmacological tool to prevent atherosclerosis and CVD.

Epidemiological studies suggest that the consumption of wine, particularly of red wine, reduces the incidence of mortality and morbidity from coronary heart disease. This has given rise to what is now popularly termed the “French paradox” [107]. The cardioprotective effect of red wine has been attributed to antioxidants present in the polyphenol fraction. Grapes contain a variety of antioxidants, including resveratrol, catechin, epicatechin, and proanthocyanidins. Of these, recent data provide interesting insights into the effect of resveratrol on the life span of simple eukaryotes such as yeast and flies by activating the longevity genes such as AMPK, SIRT1, PGC-1*α*, and NRF and has been suggested as a CR mimetic [108–111]. 

Emerging evidence indicates that resveratrol increases mitochondrial biogenesis and reduces oxidative stress in a wide variety of age-associated disease models. It has been demonstrated that resveratrol induces mitochondrial biogenesis and ameliorates angiotensin II-induced cardiac remodeling through the activation of SIRT1, PGC-1*α*, and NRF [112]. Csiszar et al. [113] demonstrated that resveratrol induced activation of PGC-1*α* and NRF, increased mitochondrial mass and mitochondrial DNA content, and upregulated protein expression of the ETC constituents in endothelial cells. Mitochondrial biogenesis induced by resveratrol treatment also has important impact on the liver and brain functions and seems to have significant consequences in the skeletal muscle, because resveratrol-treated mice exhibit an approximately twofold increase in endurance exercise [114]. Future studies are warranted to investigate whether resveratrol or specific activators of AMPK, SIRT1, PGC-1*α*, or NRF each alone or in combination can ameliorate age-associated diseases and prolong healthy life span in human subjects.

### 7.2. Upregulation of Uncoupling Proteins

Uncoupling proteins (UCPs) are mitochondrial transporters present in the inner membrane of mitochondria. The term “uncoupling protein” was originally used for UCP1, which is uniquely present in mitochondria of brown adipocytes, the thermogenic cells that maintain body temperature [115]. In these cells, UCP1 acts as a proton carrier activated by free fatty acids and creates a shunt between complexes of the respiratory chain and ATP synthase. Activation of UCP1 enhances mitochondrial respiration, and the uncoupling process results in a futile cycle and dissipation of oxidation energy as heat. In comparison to the established uncoupling and thermogenic activities of UCP1, UCP2 and UCP3 appear to be involved in the limitation of ROS levels in cells rather than in physiological uncoupling and thermogenesis [116, 117]. UCP2 and UCP3 dissipate electrochemical gradient produced by complex I, III, and IV in the mitochondrial ETC to pump protons outside the inner membrane. Such partial uncoupling of respiration prevents an exaggerated increase in ATP level that would lead to stagnation of electrons and increases their leakage from the ETC to produce superoxide. Consistent with this notion is the fact that low concentrations of chemical uncoupling agents attenuate mitochondrial ROS production [118]. Thus, UCP3 exerts an antioxidant function selectively at times when proton motive force is high, such as in resting muscle (low ATP demand), or when activity of the ETC enzyme complexes is inhibited, such as during hypoxia [119]. 

 The mechanism by which UCPs are activated appears to involve redox signaling through mild uncoupling and membrane depolarization of mitochondria that feedbacks to prevent robust ROS generation. It has been demonstrated that superoxide activates UCP3 in rat skeletal muscle mitochondria [120]. Brand and associates [121] also reported activation of UCP3 by 4-hydroxynonenal, a by-product of lipid peroxidation. Furthermore, UCP2 and UCP3 are acutely activated by ROS generated by mitochondria, which then directly modulate the glutathionylation status of the UCP to decrease ROS emission [122]. This observation indicates that GSH/GSSG ratio within mitochondria may determine the activity of UCPs and lend further support to the hypothesis that redox signaling through mild oxidative stress enhances intrinsic antioxidative capacity of mitochondria.

The physiological signals that modulate UCP3 gene expression include fasting [123, 124], acute exercise [125, 126], and increased fatty acid intake [127, 128]. Peroxisome proliferator-activated receptor (PPAR-*γ*) agonist thiazolidinedione (TZD) or PPAR-*α* agonist WY-14643 increased UCP gene expression in the brown adipose tissue [129]. PPAR-*α* induction of UCP2 protected against elevated ROS during drug-induced hepatotoxicity [130]. UCP3 expression has been shown to be increased by PPAR-*α* agonist WY-14643 in the rodent hearts [131, 132]. It has been demonstrated that activation of PPAR-*γ* upregulated mitochondrial UCP2 expression, which decreased overproduction of ROS, improved mitochondrial ETC function at the site of complex I, and inhibited the mitochondrial apoptotic cascade leading to neuronal cell death in the hippocampus following status epilepticus [133]. 

PPAR signaling pathways are known to exert anti-inflammatory effects and attenuate atherosclerosis formation. The TZDs are the agonist for PPAR-*γ* and promote insulin sensitization and improve dyslipidemia in patients with type 2 DM, although it is unknown how PPAR agonists-induced upregulation of UCPs contributes to their beneficial effects on CVD. The PPAR-*γ* agonist rosiglitazone exerted a significant vascular protective effect in hypercholesterolemic rabbits, most likely by attenuation of oxidative and nitrosative stresses [134]. However, recent studies have shown that chronic rosiglitazone administration is associated with an increased risk of heart failure, acute MI, and death as a result of cardiovascular complications [135, 136]. Currently, exact mechanisms by which rosiglitazone exerted an adverse cardiovascular effect remain unclear. 

Although CR is the fundamental approach to upregulate PPARs [137], a variety of recently emerging drugs, in addition to TZDs, which are used for the management of life style-related diseases are known to act as PPAR agonists. Fibrates are the representative PPAR-*α* agonist that stimulates the oxidation of free fatty acids in the liver, diverting them away from triglyceride synthesis and thus reducing the hepatic synthesis of triglyceride-rich lipoproteins [138]. Fibrates can also activate PPAR-*γ* by increasing the adiponectin level [139]. The fact that the cardioprotective properties of fibrates may be largely independent of their effects on plasma lipid levels, especially in subjects with features of the metabolic syndrome [140], suggests that the increase in adiponectin may contribute to the protective effect of fibrates against CVD. Recently, some sort of ARBs such as telmisartan and to a lesser extent irbesartan partially activate PPAR-*γ* and effectively treat insulin resistance and dyslipidemia without the toxicity sometimes associated with full PPAR-*γ* agonists [141, 142]. Statins can also enhance PPAR-*γ* activation, which may at least in part be involved in their antioxidant or anti-inflammatory potential [139]. 

## 8. Conclusion

**Figure 1:**
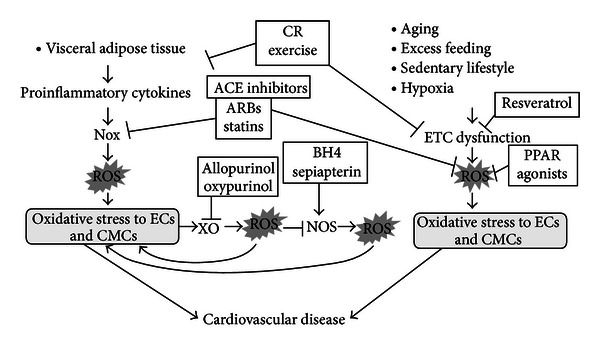
Strategy of the site-specific antioxidative therapy to prevent cardiovascular disease. The visceral adipose tissues are the primary source of proinflammatory cytokines that impose oxidative stress on endothelial cells (ECs) and cardiomyocytes (CMCs) through activation of NADPH oxidase (Nox) and generation of reactive oxygen species (ROS). Oxidative stress to ECs and CMCs then activates xanthine oxidase (XO) that potentiates the generation of ROS and increases oxidative stress to ECs and CMCs. Oxidative stress to ECs and CMCs causes uncoupling of nitric oxide synthase (NOS) through oxidation and depletion of BH_4_ that further increases oxidative stress to ECs and CMCs, creating a self-perpetuating cycle for oxidative stress in ECs and CMCs, leading to the development of cardiovascular disease. Calorie restriction (CR) or regular endurance exercise reduces the mass of the visceral adipose tissue, thereby decreasing the production of proinflammatory cytokines responsible for oxidative stress to ECs and CMCs. Angiotensin converting enzyme (ACE) inhibitors, angiotensin type 1 receptor blockers (ARBs), or statins prevents activation of Nox and mitigates oxidative stress to ECs and CMCs. Allopurinol or oxypurinol blocks XO, while tetrahydrobiopterin (BH_4_) or sepiapterin prevents uncoupling of NOS, thereby attenuating oxidative stress to ECs and CMCs. Another important source of ROS within ECs and CMCs is the electron transfer chain (ETC) in mitochondria. Aging, excess feeding, sedentary lifestyle, or hypoxia increases the leakage of electrons from the ETC and generation of ROS that imposes oxidative stress on ECs and CMCs. CR and exercise are a fundamental approach to prevent oxidative stress to ECs and CMCs by reducing the leakage of electrons from the ETC and ROS production. Resveratrol mimics the action of CR and exercise, thereby ameliorating oxidative stress to ECs and CMCs. Peroxisome proliferator-activated receptor (PPAR) *α* or *γ* agonists induce activation of uncoupling proteins in mitochondria, thereby reducing the leakage of electrons from the ETC and ROS production. ARBs and statins act as the partial agonists of PPARs that may be related to their preferable cardiovascular effects.

 Oxidative stress to endothelial cells plays a central role in the development of atherosclerosis and the occurrence of CVD. CR and endurance regular exercise are the fundamental approach to prevent oxidative stress to endothelial cells and prolong healthy life span. In addition to the improvement of lifestyle, recently emerging drugs that are effective in treating CVD have a property to eliminate ROS with a site-specific manner without interrupting favorable redox signaling, thereby ameliorating oxidative stress to endothelial cells. CR and exercise mimetics will also be fascinating pharmacological tools that specifically mitigate oxidative stress to endothelial cells. The sources of ROS and their inhibitors as well as a strategy of the site-specific antioxidative therapy to prevent CVD are presented in Table 1 and Figure 1. Further studies are warranted to establish a more efficacious approach to eliminate oxidative stress to endothelial cells and prevent atherosclerosis.

## Figures and Tables

**Table 1:** Inhibitors of ROS sources.

ROS sources	Inhibitors	Mechanisms
NADPH oxidase (Nox)	Calorie restriction exercise	(i) Reduction of visceral adipose tissue-derived inflammatory cytokines (ii) Downregulation of AT1R
	ARBs	AT1R-induced activation of Nox
	Statins	Inhibition of ROCK

Xanthine oxidase (XO)	Calorie restriction exercise	(i) Inhibition of Nox-mediated EC damage (ii) Inhibition of Nox-mediated EC damage
	ARBs	Inhibition of Nox-mediated EC damage
	Allopurinol oxypurinol	Direct inhibition of XO

	Calorie restriction exercise	Inhibition of oxidative stress and NOS uncoupling
Nitric oxide synthase (NOS)	ARBs Statins Allopurinol and oxypurinol	Inhibition of oxidative stress and NOS uncoupling
	BH4 Sepiapterin	Direct inhibition of NOS uncoupling

	Calorie restriction exercise	(i) Inhibition of electron leakage from ETC (ii) Stimulation of mitochondrial biogenesis (iii) Induction of UPs by activating PPARs
	Resveratrol	Stimulation of mitochondrial biogenesis
	TZD	Activation of PPAR-*γ* and induction of UP2 and UP3
Mitochondrial electron transfer chain (ETC)	WY-14643	Activation of PPAR-*α* and induction of UP2 and UP3
	Fibrates	Activation of PPARs and induction of UP2 and UP3
	ARBs	Activation of PPAR-*γ* and induction of UP2 and UP3
	Statins	Activation of PPAR-*γ* and induction of UP2 and UP3

AT1R: angiotensin II type 1 receptor, ARBs: angiotensin II type 1 receptor blockers, ROCK: Rho-kinase, ECs: endothelial cells, CMCs: cardiomyocytes, UP: uncoupling protein, PPAR: peroxisome proliferator-activated receptor, TZD: thiazolidinedione.

## References

[1] Sohal RS, Orr WC (2012). The redox stress hypothesis of aging. *Free Radical Biology and Medicine*.

[2] Liu Y, Fiskum G, Schubert D (2002). Generation of reactive oxygen species by the mitochondrial electron transport chain. *Journal of Neurochemistry*.

[3] Choksi KB, Nuss JE, DeFord JH, Papaconstantinou J (2011). Mitochondrial electron transport chain functions in long-lived Ames dwarf mice. *Aging*.

[4] Rueckschloss U, Duerrschmidt N, Morawietz H (2003). NADPH oxidase in endothelial cells: impact on atherosclerosis. *Antioxidants and Redox Signaling*.

[5] Hajer GR, van Haeften TW, Visseren FLJ (2008). Adipose tissue dysfunction in obesity, diabetes, and vascular diseases. *European Heart Journal*.

[6] Otani H (2011). Oxidative stress as pathogenesis of cardiovascular risk associated with metabolic syndrome. *Antioxidants & Redox Signaling*.

[7] Murdoch CE, Alom-Ruiz SP, Wang M (2011). Role of endothelial Nox2 NADPH oxidase in angiotensin II-induced hypertension and vasomotor dysfunction. *Basic Research in Cardiology*.

[8] Yokoyama M, Inoue N (2004). How vascular NAD(P)H oxidase activity and Nox isoform expression are regulated. *Arteriosclerosis, Thrombosis, and Vascular Biology*.

[9] Yang YM, Huang A, Kaley G, Sun D (2009). eNOS uncoupling and endothelial dysfunction in aged vessels. *American Journal of Physiology*.

[10] Förstermann U, Münzel T (2006). Endothelial nitric oxide synthase in vascular disease: from marvel to menace. *Circulation*.

[11] Oak JH, Cai H (2007). Attenuation of angiotensin II signaling recouples eNOS and inhibits nonendothelial NOX activity in diabetic mice. *Diabetes*.

[12] Nedeljkovic ZS, Gokce N, Loscalzo J (2003). Mechanisms of oxidative stress and vascular dysfunction. *Postgraduate Medical Journal*.

[13] Guthikonda S, Sinkey C, Barenz T, Haynes WG (2003). Xanthine oxidase inhibition reverses endothelial dysfunction in heavy smokers. *Circulation*.

[14] Rapola JM, Virtamo J, Ripatti S (1997). Randomised trial of *α*-tocopherol and *β*-carotene supplements on incidence of major coronary events in men with previous myocardial infarction. *The Lancet*.

[15] Siems W, Sommerburg O, Schild L, Augustin W, Langhans CD, Wiswedel I (2002). Beta-carotene cleavage products induce oxidative stress in vitro by impairing mitochondrial respiration. *The FASEB Journal*.

[16] Kidd P (2011). Astaxanthin, cell membrane nutrient with diverse clinical benefits and anti-aging potential. *Alternative Medicine Review*.

[17] Murry CE, Jennings RB, Reimer KA (1986). Preconditioning with ischemia: a delay of lethal cell injury in ischemic myocardium. *Circulation*.

[18] Bolli R (2000). The late phase of preconditioning. *Circulation Research*.

[19] Otani H (2008). Ischemic preconditioning: from molecular mechanisms to therapeutic opportunities. *Antioxidants and Redox Signaling*.

[20] Baines CP, Goto M, Downey JM (1997). Oxygen radicals released during ischemic preconditioning contribute to cardioprotection in the rabbit myocardium. *Journal of Molecular and Cellular Cardiology*.

[21] Chen W, Gabel S, Steenbergen C, Murphy E (1995). A redox-based mechanism for cardioprotection induced by ischemic preconditioning in perfused rat heart. *Circulation Research*.

[22] Tritto I, D’Andrea D, Eramo N (1997). Oxygen radicals can induce preconditioning in rabbit hearts. *Circulation Research*.

[23] Otani H (2004). Reactive oxygen species as mediators of signal transduction in ischemic preconditioning. *Antioxidants and Redox Signaling*.

[24] Yamashita N, Hoshida S, Taniguchi N, Kuzuya T, Hori M (1998). Whole-body hyperthermia provides biphasic cardioprotection against ischemia/reperfusion injury in the rat. *Circulation*.

[25] Bedard K, Krause KH (2007). The NOX family of ROS-generating NADPH oxidases: physiology and pathophysiology. *Physiological Reviews*.

[26] Nauseef WM (2008). Biological roles for the NOX family NADPH oxidases. *The Journal of Biological Chemistry*.

[27] Touyz RM, Chen X, Tabet F (2002). Expression of a functionally active gp91phox-containing neutrophil-type NAD(P)H oxidase in smooth muscle cells from human resistance arteries: regulation by angiotensin II. *Circulation Research*.

[28] Hattori Y, Akimoto K, Gross SS, Hattori S, Kasai K (2005). Angiotensin-II-induced oxidative stress elicits hypoadiponectinaemia in rats. *Diabetologia*.

[29] Li JM, Shah AM (2003). Mechanism of endothelial cell NADPH oxidase activation by angiotensin II. role of the p47phox subunit. *The Journal of Biological Chemistry*.

[30] Ray R, Murdoch CE, Wang M (2011). Endothelial Nox4 NADPH oxidase enhances vasodilatation and reduces blood pressure in vivo. *Arteriosclerosis, Thrombosis, and Vascular Biology*.

[31] Manrique C, Lastra G, Gardner M, Sowers JR (2009). The renin angiotensin aldosterone system in hypertension: roles of insulin resistance and oxidative stress. *Medical Clinics of North America*.

[32] Sata M, Fukuda D (2010). Crucial role of renin-angiotensin system in the pathogenesis of atherosclerosis. *Journal of Medical Investigation*.

[33] Boyle JJ (2005). Macrophage activation in atherosclerosis: pathogenesis and pharmacology of plaque rupture. *Current Vascular Pharmacology*.

[34] Wang G, Woo CWH, Sung FL (2002). Increased monocyte adhesion to aortic endothelium in rats with hyperhomocysteinemia: role of chemokine and adhesion molecules. *Arteriosclerosis, Thrombosis, and Vascular Biology*.

[35] Zhang W, Elimban V, Nijjar MS, Gupta SK, Dhalla NS (2003). Role of mitogen-activated protein kinase in cardiac hypertrophy and heart failure. *Experimental and Clinical Cardiology*.

[36] Mehta PK, Griendling KK (2007). Angiotensin II cell signaling: physiological and pathological effects in the cardiovascular system. *American Journal of Physiology*.

[37] Zhao W, Zhao T, Chen Y, Ahokas RA, Sun Y (2008). Oxidative stress mediates cardiac fibrosis by enhancing transforming growth factor-beta1 in hypertensive rats. *Molecular and Cellular Biochemistry*.

[38] Berg AH, Scherer PE (2005). Adipose tissue, inflammation, and cardiovascular disease. *Circulation Research*.

[39] El-Swefy S, Schaefer EJ, Seman LJ (2000). The effect of vitamin E, probucol, and lovastatin on oxidative status and aortic fatty lesions in hyperlipidemic-diabetic hamsters. *Atherosclerosis*.

[40] Rikitake Y, Liao JK (2005). Rho GTPases, statins, and nitric oxide. *Circulation Research*.

[41] Hordijk PL (2006). Regulation of NADPH oxidases: the role of Rac proteins. *Circulation Research*.

[42] Bendall JK, Cave AC, Heymes C, Gall N, Shah AM (2002). Pivotal role of a gp91(phox)-containing NADPH oxidase in angiotensin II-induced cardiac hypertrophy in mice. *Circulation*.

[43] Kang BY, Mehta JL (2009). Rosuvastatin attenuates Ang II—mediated cardiomyocyte hypertrophy via inhibition of LOX-1. *Journal of Cardiovascular Pharmacology and Therapeutics*.

[44] Zhou Q, Liao JK (2009). Rho kinase: an important mediator of atherosclerosis and vascular disease. *Current Pharmaceutical Design*.

[45] Szostak J, Laurant P (2011). The forgotten face of regular physical exercise: a ‘natural’ anti-atherogenic activity. *Clinical Science*.

[46] Pacher P, Nivorozhkin A, Szabó C (2006). Therapeutic effects of xanthine oxidase inhibitors: renaissance half a century after the discovery of allopurinol. *Pharmacological Reviews*.

[47] Campion EW, Glynn RJ, DeLabry LO (1987). Asymptomatic hyperuricemia. Risks and consequences in the Normative Aging Study. *The American Journal of Medicine*.

[48] Li JM, Shah AM (2004). Endothelial cell superoxide generation: regulation and relevance for cardiovascular pathophysiology. *American Journal of Physiology*.

[49] Harrison D, Griendling KK, Landmesser U, Hornig B, Drexler H (2003). Role of oxidative stress in atherosclerosis. *American Journal of Cardiology*.

[50] Madamanchi NR, Vendrov A, Runge MS (2005). Oxidative stress and vascular disease. *Arteriosclerosis, Thrombosis, and Vascular Biology*.

[51] Bonomini F, Tengattini S, Fabiano A, Bianchi R, Rezzani R (2008). Atherosclerosis and oxidative stress. *Histology and Histopathology*.

[52] Landmesser U, Spiekermann S, Preuss C (2007). Angiotensin II induces endothelial xanthine oxidase activation: role for endothelial dysfunction in patients with coronary disease. *Arteriosclerosis, Thrombosis, and Vascular Biology*.

[53] McNally JS, Davis ME, Giddens DP (2003). Role of xanthine oxidoreductase and NAD(P)H oxidase in endothelial superoxide production in response to oscillatory shear stress. *American Journal of Physiology*.

[54] Li PF, Dietz R, von Harsdorf R (1997). Differential effect of hydrogen peroxide and superoxide anion on apoptosis and proliferation of vascular smooth muscle cells. *Circulation*.

[55] Zhang C, Xu X, Potter BJ (2006). TNF-*α* contributes to endothelial dysfunction in ischemia/reperfusion injury. *Arteriosclerosis, Thrombosis, and Vascular Biology*.

[56] Gao X, Zhang H, Belmadani S (2008). Role of TNF-*α*-induced reactive oxygen species in endothelial dysfunction during reperfusion injury. *American Journal of Physiology*.

[57] Kleinbongard P, Schulz R, Heusch G (2011). TNF*α* in myocardial ischemia/reperfusion, remodeling and heart failure. *Heart Failure Reviews*.

[58] Yamamoto Y, Ogino K, Igawa G (2006). Allopurinol reduces neointimal hyperplasia in the carotid artery ligation model in spontaneously hypertensive rats. *Hypertension Research*.

[59] Kushiyama A, Okubo H, Sakoda H, Kikuchi T, Fujishiro M, Sato H (2012). Xanthine oxidoreductase is involved in macrophage foam cell formation and atherosclerosis development. *Arteriosclerosis, Thrombosis, and Vascular Biology*.

[60] Dogan A, Yarlioglues M, Kaya MG (2011). Effect of long-term and high-dose allopurinol therapy on endothelial function in normotensive diabetic patients. *Blood Pressure*.

[61] Doehner W, Schoene N, Rauchhaus M (2002). Effects of xanthine oxidase inhibition with allopurinol on endothelial function and peripheral blood flow in hyperuricemic patients with chronic heart failure: results from 2 placebo-controlled studies. *Circulation*.

[62] Manning AS, Coltart DJ, Hearse DJ (1984). Ischemia and reperfusion-induced arrhythmias in the rat. Effects of xanthine oxidase inhibition with allopurinol. *Circulation Research*.

[63] Ignarro LJ (1990). Nitric oxide. A novel signal transduction mechanism for transcellular communication. *Hypertension*.

[64] Moncada S, Palmer RMJ, Higgs EA (1991). Nitric oxide: physiology, pathophysiology, and pharmacology. *Pharmacological Reviews*.

[65] Palmer RMJ, Ferrige AG, Moncada S (1987). Nitric oxide release accounts for the biological activity of endothelium-derived relaxing factor. *Nature*.

[66] Mayer B, Hemmens B (1997). Biosynthesis and action of nitric oxide in mammalian cells. *Trends in Biochemical Sciences*.

[67] Stuehr D, Pou S, Rosen GM (2001). Oxygen reduction by nitric-oxide synthases. *The Journal of Biological Chemistry*.

[68] Abu-Soud HM, Stuehr DJ (1993). Nitric oxide synthases reveal a role for calmodulin in controlling electron transfer. *Proceedings of the National Academy of Sciences of the United States of America*.

[69] Moens AL, Kass DA (2006). Tetrahydrobiopterin and cardiovascular disease. *Arteriosclerosis, Thrombosis, and Vascular Biology*.

[70] Moens AL, Kass DA (2007). Therapeutic potential of tetrahydrobiopterin for treating vascular and cardiac disease. *Journal of Cardiovascular Pharmacology*.

[71] Tarpey MM (2002). Sepiapterin treatment in atherosclerosis. *Arteriosclerosis, Thrombosis, and Vascular Biology*.

[72] Vásquez-Vivar J, Duquaine D, Whitsett J, Kalyanaraman B, Rajagopalan S (2002). Altered tetrahydrobiopterin metabolism in atherosclerosis: implications for use of oxidized tetrahydrobiopterin analogues and thiol antioxidants. *Arteriosclerosis, Thrombosis, and Vascular Biology*.

[73] Vasquez-Vivar J, Martasek P, Whitsett J, Joseph J, Kalyanaraman B (2002). The ratio between tetrahydrobiopterin and oxidized tetrahydrobiopterin analogues controls superoxide release from endothelial nitric oxide synthase: an EPR spin trapping study. *Biochemical Journal*.

[74] Thöny B, Auerbach G, Blau N (2000). Tetrahydrobiopterin biosynthesis, regeneration and functions. *Biochemical Journal*.

[75] Fiege B, Ballhausen D, Kierat L (2004). Plasma tetrahydrobiopterin and its pharmacokinetic following oral administration. *Molecular Genetics and Metabolism*.

[76] Shi W, Meininger CJ, Haynes TE, Hatakeyama K, Wu G (2004). Regulation of tetrahydrobiopterin synthesis and bioavailability in endothelial cells. *Cell Biochemistry and Biophysics*.

[77] Wang X, Hattori Y, Satoh H (2007). Tetrahydrobiopterin prevents endothelial dysfunction and restores adiponectin levels in rats. *European Journal of Pharmacology*.

[78] Shinozaki K, Nishio Y, Yoshida Y (2005). Supplement of tetrahydrobiopterin by a gene transfer of GTP cyclohydrolase I cDNA improves vascular dysfunction in insulin-resistant rats. *Journal of Cardiovascular Pharmacology*.

[79] Katusic ZS, d’Uscio LV, Nath KA (2009). Vascular protection by tetrahydrobiopterin: progress and therapeutic prospects. *Trends in Pharmacological Sciences*.

[80] Heitzer T, Brockhoff C, Mayer B (2000). Tetrahydrobiopterin improves endothelium-dependent vasodilation in chronic smokers: evidence for a dysfunctional nitric oxide synthase. *Circulation Research*.

[81] Sayed N, Kim DD, Fioramonti X, Iwahashi T, Durán WN, Beuve A (2008). Nitroglycerin-induced S-nitrosylation and desensitization of soluble guanylyl cyclase contribute to nitrate tolerance. *Circulation Research*.

[82] Gonzalez DR, Beigi F, Treuer AV, Hare JM (2007). Deficient ryanodine receptor S-nitrosylation increases sarcoplasmic reticulum calcium leak and arrhythmogenesis in cardiomyocytes. *Proceedings of the National Academy of Sciences of the United States of America*.

[83] Lim G, Venetucci L, Eisner DA, Casadei B (2008). Does nitric oxide modulate cardiac ryanodine receptor function? Implications for excitation-contraction coupling. *Cardiovascular Research*.

[84] Shimazu T, Otani H, Yoshioka K, Fujita M, Okazaki T, Iwasaka T (2011). Sepiapterin enhances angiogenesis and functional recovery in mice after myocardial infarction. *American Journal of Physiology*.

[85] Okazaki T, Otani H, Shimazu T (2011). Reversal of inducible nitric oxide synthase uncoupling unmasks tolerance to ischemia/reperfusion injury in the diabetic rat heart. *Journal of Molecular and Cellular Cardiology*.

[86] Sun J, Morgan M, Shen RF, Steenbergen C, Murphy E (2007). Preconditioning results in S-nitrosylation of proteins involved in regulation of mitochondrial energetics and calcium transport. *Circulation Research*.

[87] Kirkinezos IG, Moraes CT (2001). Reactive oxygen species and mitochondrial diseases. *Seminars in Cell and Developmental Biology*.

[88] Verdejo HE, del Campo A, Troncoso R, Gutierrez T, Toro B, Quiroga C (2012). Mitochondria, myocardial remodeling, and cardiovascular disease. *Current Hypertension Reports*.

[89] Chance B, Sies H, Boveris A (1979). Hydroperoxide metabolism in mammalian organs. *Physiological Reviews*.

[90] Hansford RG, Hogue BA, Mildaziene V (1997). Dependence of H_2_O_2_ formation by rat heart mitochondria on substrate availability and donor age. *Journal of Bioenergetics and Biomembranes*.

[91] Wallace DC (2005). A mitochondrial paradigm of metabolic and degenerative diseases, aging, and cancer: a dawn for evolutionary medicine. *Annual Review of Genetics*.

[92] Gomes EC, Silva AN, de Oliveira MR (2012). Oxidants, antioxidants, and the beneficial roles of exercise-induced production of reactive species. *Oxidative Medicine and Cellular Longevity*.

[93] Herlein JA, Fink BD, Henry DM, Yorek MA, Teesch LM, Sivitz WI (2011). Mitochondrial superoxide and coenzyme Q in insulin-deficient rats: increased electron leak. *American Journal of Physiology*.

[94] Jastroch M, Divakaruni AS, Mookerjee S, Treberg JR, Brand MD (2010). Mitochondrial proton and electron leaks. *Essays in Biochemistry*.

[95] Reznick RM, Shulman GI (2006). The role of AMP-activated protein kinase in mitochondrial biogenesis. *The Journal of Physiology*.

[96] Viollet B, Guigas B, Leclerc J (2009). AMP-activated protein kinase in the regulation of hepatic energy metabolism: from physiology to therapeutic perspectives. *Acta Physiologica*.

[97] Yassine HN, Marchetti CM, Krishnan RK, Vrobel TR, Gonzalez F, Kirwan JP (2009). Effects of exercise and caloric restriction on insulin resistance and cardiometabolic risk factors in older obese adults—a randomized clinical trial. *Journals of Gerontology A*.

[98] Cantó C, Auwerx J (2009). PGC-1*α*, SIRT1 and AMPK, an energy sensing network that controls energy expenditure. *Current Opinion in Lipidology*.

[99] Valle I, Álvarez-Barrientos A, Arza E, Lamas S, Monsalve M (2005). PGC-1*α* regulates the mitochondrial antioxidant defense system in vascular endothelial cells. *Cardiovascular Research*.

[100] Irrcher I, Ljubicic V, Hood DA (2009). Interactions between ROS and AMP kinase activity in the regulation of PGC-1*α* transcription in skeletal muscle cells. *American Journal of Physiology*.

[101] Lira VA, Brown DL, Lira AK (2010). Nitric oxide and AMPK cooperatively regulate PGC-1*α* in skeletal muscle cells. *The Journal of Physiology*.

[102] Nisoli E, Clementi E, Paolucci C (2003). Mitochondrial biogenesis in mammals: the role of endogenous nitric oxide. *Science*.

[103] Piantadosi CA, Suliman HB (2006). Mitochondrial transcription factor A induction by redox activation of nuclear respiratory factor 1. *The Journal of Biological Chemistry*.

[104] Kukidome D, Nishikawa T, Sonoda K (2006). Activation of AMP-activated protein kinase reduces hyperglycemia-induced mitochondrial reactive oxygen species production and promotes mitochondrial biogenesis in human umbilical vein endothelial cells. *Diabetes*.

[105] Scarpulla RC, Vega RB, Kelly DP (2012). Transcriptional integration of mitochondrial biogenesis. *Trends in Endocrinology & Metabolism*.

[106] Scarpulla RC (2008). Transcriptional paradigms in mammalian mitochondrial biogenesis and function. *Physiological Reviews*.

[107] Constant J (1997). Alcohol, ischemic heart disease, and the French paradox. *Coronary Artery Disease*.

[108] Dasgupta B, Milbrandt J (2007). Resveratrol stimulates AMP kinase activity in neurons. *Proceedings of the National Academy of Sciences of the United States of America*.

[109] Howitz KT, Bitterman KJ, Cohen HY (2003). Small molecule activators of sirtuins extend *Saccharomyces cerevisiae* lifespan. *Nature*.

[110] Swindell WR (2008). Comparative analysis of microarray data identifies common responses to caloric restriction among mouse tissues. *Mechanisms of Ageing and Development*.

[111] Wood JG, Rogina B, Lavu S (2004). Sirtuin activators mimic caloric restriction and delay ageing in metazoans. *Nature*.

[112] Biala A, Tauriainen E, Siltanen A (2010). Resveratrol induces mitochondrial biogenesis and ameliorates Ang II-induced cardiac remodeling in transgenic rats harboring human renin and angiotensinogen genes. *Blood Pressure*.

[113] Csiszar A, Labinskyy N, Pinto JT (2009). Resveratrol induces mitochondrial biogenesis in endothelial cells. *American Journal of Physiology*.

[114] Ungvari Z, Sonntag WE, de Cabo R, Baur JA, Csiszar A (2011). Mitochondrial protection by resveratrol. *Exercise and Sport Sciences Reviews*.

[115] Matthias A, Ohlson KBE, Fredriksson JM, Jacobsson A, Nedergaard J, Cannon B (2000). Thermogenic responses in brown fat cells are fully UCP1-dependent. UCP2 or UCP3 do not substitute for UCP1 in adrenergically or fatty acid-induced thermogenesis. *The Journal of Biological Chemistry*.

[116] Nègre-Salvayre A, Hirtz C, Carrera G (1997). A role for uncoupling protein-2 as a regulator of mitochondrial hydrogen peroxide generation. *The FASEB Journal*.

[117] Rousset S, Alves-Guerra MC, Mozo J (2004). The biology of mitochondrial uncoupling proteins. *Diabetes*.

[118] Korshunov SS, Skulachev VP, Starkov AA (1997). High protonic potential actuates a mechanism of production of reactive oxygen species in mitochondria. *FEBS Letters*.

[119] Chandel N, Budinger GRS, Kemp RA, Schumacker PT (1995). Inhibition of cytochrome-c oxidase activity during prolonged hypoxia. *American Journal of Physiology*.

[120] Echtay KS, Roussel D, St-Plerre J (2002). Superoxide activates mitochondrial uncoupling proteins. *Nature*.

[121] Parker N, Vidal-Puig A, Brand MD (2008). Stimulation of mitochondrial proton conductance by hydroxynonenal requires a high membrane potential. *Bioscience Reports*.

[122] Mailloux RJ, Harper ME (2011). Uncoupling proteins and the control of mitochondrial reactive oxygen species production. *Free Radical Biology and Medicine*.

[123] Boss O, Samec S, Kühne F (1998). Uncoupling protein-3 expression in rodent skeletal muscle is modulated by food intake but not by changes in environmental temperature. *The Journal of Biological Chemistry*.

[124] Millet L, Vidal H, Andreelli F (1997). Increased uncoupling protein-2 and -3 mRNA expression during fasting in obese and lean humans. *The Journal of Clinical Investigation*.

[125] Tsuboyama-Kasaoka N, Tsunoda N, Maruyama K (1998). Up-regulation of uncoupling protein 3 (UCP3) mRNA by exercise training and down-regulation of UCP3 by denervation in skeletal muscles. *Biochemical and Biophysical Research Communications*.

[126] Schrauwen P, Hesselink MKC, Vaartjes I (2002). Effect of acute exercise on uncoupling protein 3 is a fat metabolism-mediated effect. *American Journal of Physiology*.

[127] Matsuda J, Hosoda K, Itoh H (1997). Cloning of rat uncoupling protein-3 and uncoupling protein-2 cDNAs: their gene expression in rats fed high-fat diet. *FEBS Letters*.

[128] Schrauwen P, Hoppeler H, Billeter R, Bakker AHF, Pendergast DR (2001). Fiber type dependent upregulation of human skeletal muscle UCP2 and UCP3 mRNA expression by high-fat diet. *International Journal of Obesity*.

[129] Kelly LJ, Vicario PP, Thompson GM (1998). Peroxisome proliferator-activated receptors *γ* and *α* mediate in vivo regulation of uncoupling protein (UCP-1, UCP-2, UCP-3) gene expression. *Endocrinology*.

[130] Patterson AD, Shah YM, Matsubara T, Krausz KW, Gonzalez FJ (2012). Peroxisome proliferator-activated receptor alpha induction of uncoupling protein 2 protects against acetaminophen-induced liver toxicity. *Hepatology*.

[131] Young ME, Patil S, Ying J (2001). Uncoupling protein 3 transcription is regulated by peroxisome proliferator-activated receptor *α* in the adult rodent heart. *The FASEB Journal*.

[132] Murray AJ, Panagia M, Hauton D, Gibbons GF, Clarke K (2005). Plasma free fatty acids and peroxisome proliferator-activated receptor *α* in the control of myocardial uncoupling protein levels. *Diabetes*.

[133] Chuang YC, Lin TK, Huang HY (2012). Peroxisome proliferator-activated receptors gamma/mitochondrial uncoupling protein 2 signaling protects against seizure-induced neuronal cell death in the hippocampus following experimental status epilepticus. *Journal of Neuroinflammation*.

[134] Tao L, Liu HR, Gao E (2003). Antioxidative, antinitrative, and vasculoprotective effects of a peroxisome proliferator-activated receptor-*γ* agonist in hypercholesterolemia. *Circulation*.

[135] Nissen SE, Wolski K (2007). Effect of rosiglitazone on the risk of myocardial infarction and death from cardiovascular causes. *The New England Journal of Medicine*.

[136] Psaty BM, Furberg CD (2007). Rosiglitazone and cardiovascular risk. *The New England Journal of Medicine*.

[137] Masternak MM, Bartke A (2007). PPARs in calorie restricted and genetically long-lived mice. *PPAR Research*.

[138] Staels B, Dallongeville J, Auwerx J, Schoonjans K, Leitersdorf E, Fruchart JC (1998). Mechanism of action of fibrates on lipid and lipoprotein metabolism. *Circulation*.

[139] Perelas A, Tsoulkani A, Perrea D (2010). Effects of lipid-lowering drugs on adiponectin. *Current Vascular Pharmacology*.

[140] Barter PJ, Rye KA (2008). Is there a role for fibrates in the management of dyslipidemia in the metabolic syndrome?. *Arteriosclerosis, Thrombosis, and Vascular Biology*.

[141] Tuck ML (2005). Angiotensin-receptor blocking agents and the peroxisome proliferator-activated receptor-*γ* system. *Current Hypertension Reports*.

[142] Towfighi A, Ovbiagele B (2008). Partial peroxisome proliferator-activated receptor agonist angiotensin receptor blockers. Potential multipronged strategy in stroke prevention. *Cerebrovascular Diseases*.

