# Erratum to “Site-Specific Antioxidative Therapy for Prevention of Atherosclerosis and Cardiovascular Disease”

**DOI:** 10.1155/2013/936436

**Published:** 2013-10-03

**Authors:** Hajime Otani

**Affiliations:** Internal Medicine II, Kansai Medical University, Moriguchi City, Japan

The Research Grant number in Acknowledgments section in page 9 stating that “This work was supported in part by Research Grant 20590847” was wrong and the corrected one is “23591070.”

